# High-temperature magnetism and microstructure of a semiconducting ferromagnetic (GaSb)_1−_*_x_*(MnSb)*_x_* alloy

**DOI:** 10.3762/bjnano.9.230

**Published:** 2018-09-14

**Authors:** Leonid N Oveshnikov, Elena I Nekhaeva, Alexey V Kochura, Alexander B Davydov, Mikhail A Shakhov, Sergey F Marenkin, Oleg A Novodvorskii, Alexander P Kuzmenko, Alexander L Vasiliev, Boris A Aronzon, Erkki Lahderanta

**Affiliations:** 1 National Research Center ”Kurchatov Institute”, 123182 Moscow, Russian Federation; 2 P.N. Lebedev Physical Institute, Russian Academy of Sciences, 119991 Moscow, Russian Federation; 3 South-West State University, 305040 Kursk, Russian Federation; 4 Lappeenranta University of Technology, 53850 Lappeenranta, Finland; 5 Ioffe Institute, 194021 St. Petersburg, Russian Federation; 6 Kurnakov Institute of General and Inorganic Chemistry, Russian Academy of Sciences, 119991 Moscow, Russian Federation; 7 National University of Science and Technology MISIS, 119049 Moscow, Russian Federation; 8 Institute on Laser and Information Technologies, Russian Academy of Sciences, 140700 Shatura, Moscow Region, Russian Federation

**Keywords:** anomalous Hall effect, high-temperature ferromagnetism, nanostructured materials, thin films

## Abstract

We have studied the properties of relatively thick (about 120 nm) magnetic composite films grown by pulsed laser deposition using the eutectic compound (GaSb)_0.59_(MnSb)_0.41_ as target for sputtering. For the studied films we have observed ferromagnetism and an anomalous Hall effect above room temperature, confirming the presence of spin-polarized carriers. Electron microscopy, atomic and magnetic force microscopy results suggest that the films under study have a homogenous columnar structure in the bulk while MnSb inclusions accumulate near the surface. This is in good agreement with the high mobility values of charge carriers. Based on our data we conclude that the magnetic and magnetotransport properties of the films at room temperature are defined by the MnSb inclusions.

## Introduction

Diluted magnetic semiconductors (DMS) are very promising materials for spintronic devices, because DMS offer the combination of magnetic and semiconducting properties. Currently, the most commonly studied DMS systems are those based on III–V semiconductors doped by Mn [1–3]. Among these systems, the most well-known and extensively studied is Ga_1−_*_x_*Mn*_x_*As. Here Mn atoms substitute Ga atoms and establish a ferromagnetic state realized through carrier-induced indirect exchange between Mn atoms by a Zener–RKKY mechanism accompanied by the spin polarization of conducting holes [1,3]. To reach high values of the Curie temperature, *T*_c_, materials with high Mn concentration are required, which can be achieved by using non-equilibrium growth methods, such as low-temperature molecular-beam epitaxy. Thus, the solubility limit of Mn in Ga_1−_*_x_*Mn*_x_*As can be increased up to *x* = 0.2 without precipitate formation [4]. The highest *T*_c_ values achieved in these materials was below 200 K, observed with *x* values of about 0.1 [5–6]. This is remarkably high for a DMS system while for practical applications *T*_c_ *>* 300 K is desired. At higher concentrations Mn atoms start to occupy interstitial sites and produce strong structure defects that increase the scattering of charge carriers. Thus, hole mobilities in Ga_1−_*_x_*Mn*_x_*As systems with high *T*_c_ (*x >* 0.06) usually do not exceed 10 cm^2^/(V·s).

Another way to realize high *T*_c_ values in magnetic semiconductor materials is to create a granular system with two phases, i.e., ferromagnetic nanoinclusions embedded into a semiconductor matrix. Although such systems are studied less frequently, in some of the related works the observed *T*_c_ values exceeded room temperature [7–13]. An additional advantage of granular systems are the higher values of carrier mobility, about one order of magnitude higher than that in traditional DMS such as Ga_1−_*_x_*Mn*_x_*As. This is due to the aggregation of the majority of magnetic impurity atoms within nanoinclusions, which results in a higher crystalline quality of the semiconductor matrix [9] and a lower density of scatterers. Thus, granular materials could be of interest, both as an object of fundamental studies of DMS systems and as a versatile material suitable for testing prototype spintronic devices.

Recent studies of MnAs inclusions embedded into a GaAs matrix showed that inclusions can emerge with two types of crystal structure. The magnetic properties of MnAs inclusions with zinc blende type and hexagonal lattices are substantially different. The actual application of GaAs:MnAs materials is restricted by this fact.

A second recently studied nanocomposite system is a GaSb matrix with incorporated MnSb nanograins [8–10,14]. However, the best results were obtained not for a composite system but for (GaSb)_1−_*_x_*(MnSb)*_x_* alloys with *x* = 0.41. In annealed samples the mobility of holes was about 100 cm^2^/(V·s) and *T*_c_ was above room temperature [11–12]. Earlier it was suggested [9] that the ferromagnetic ordering in this case is induced by the interaction of MnSb magnetic clusters with carriers inside the matrix. It should induce carrier spin-polarization and lead to the formation of a long-range ferromagnetic percolation cluster, which includes both MnSb magnetic clusters and spin-polarized carriers. However, to verify this assumption and to reveal the origin of the high-temperature ferromagnetism in GaSb–MnSb alloys, one needs a detailed knowledge of the sample structure.

Thus, in this paper we investigate magnetic and transport properties of the GaSb–MnSb alloy films with *x* = 0.41 and we elucidate the origin of the ferromagnetic state in this material. We use atomic force and magnetic force microscopy (AFM and MFM) as well as scanning/transmission electron microscopy (S/TEM) to study the sample structure.

## Experimental

GaSb–MnSb films with a thickness *d* in the range between 120 and 135 nm and an area of 0.1–1.0 cm^2^ were grown by droplet-free pulsed laser deposition (PLD) in high vacuum (10^−6^ Torr) with deposition temperatures of *T*_dep_ = 100–300 °C. We employed a GaSb–MnSb target of eutectic composition containing 41 mol % MnSb and 59 mol % GaSb, which was sputtered by the second harmonic radiation of a Q-switched YAG:Nd laser (λ = 532 nm). Al_2_O_3_(0001) single crystals were used as substrates. A more detailed description of the growth technology can be found in [15].

Magnetization was measured at temperatures of *T* = 5–310 K in magnetic fields up to *H* = 50 kOe using a superconducting quantum interference device (SQUID) magnetometer S600X (Cryogenic, UK). The electrical and magnetotransport properties were investigated at temperatures of *T* = 2–320 K using a standard six-probe geometry in pulsed magnetic fields up to *H* = 300 kOe. The studied samples demonstrated linear current–voltage characteristics down to sub-helium temperatures while sustaining high values of conductivity.

The cross-section specimens for S/TEM studies were prepared by focus ion beam (FIB) milling in a Helios (FEI, US) SEM/FIB dual-beam system equipped with C and Pt gas injectors and a micromanipulator (Omniprobe, US). A 2 μm Pt layer was deposited on the surface of the sample prior to the cross-section preparation by FIB milling. Sections of approximately 8 × 5 μm^2^ area and 2 μm thickness were cut by 30 kV Ga^+^ ions, removed from the sample and then attached to the Omniprobe semiring (Omniprobe, US). Final thinning was performed with 5 kV Ga^+^ ions followed by cleaning by 2 keV Ga^+^ ions for electron transparency. All specimens were studied in a scanning/transmission electron microscope Titan 80-300 (FEI, US) equipped with a spherical aberration (Cs) corrector (electron probe corrector), a high-angle annular dark field (HAADF) detector, an atmospheric thin-window energy dispersive X-ray (EDX) spectrometer (Phoenix System, EDAX, US) and a post-column Gatan energy filter (GIF), (Gatan, US). The S/TEM was operated at 300 kV. Digital micrograph (Gatan, US) and TIA software (FEI, US) was used for image analysis. P. Stadelmann’s JEMS software [16] was used for the simulation of diffraction patterns and images.

Scanning atomic force microscopy (AFM) and magnetic force microscopy (MFM) images were obtained on an SmartSPM microscope (AIST-NT, US) at temperatures of *T* = 295–450 K.

## Results and Discussion

We have studied several (GaSb)_1−_*_x_*(MnSb)*_x_* samples with *x* = 0.41, both annealed and not annealed. In this paper we discuss only samples annealed at 350 °C for 30 min with high hole concentrations of *N*_p_ *>* 10^19^ cm^−3^ because they showed much better magnetic and semiconducting (electron transport) properties. Therefore, these samples are more suitable to reveal the nature of magnetic properties and hole spin-polarization in this material, which is the aim of this paper. Main parameters of the studied samples are presented in Table 1.

**Table 1:** Sample parameters: deposition temperature *T*_dep_; film thickness *d*; carrier concentration *N*_p_; carrier mobility μ; coercive force *H*_c_; remanent magnetization *M*_rem_; saturation magnetization *M*_sat_ (*N*_p_, μ and *M*_sat_ were obtained at *T* = 300 K, while the values of *H*_c_ and *M*_rem_ correspond to *T* = 4.2 K).

sample	*T*_dep_,	*d*,	*N*_p_,	μ,	*H*_c_^a^,	*M*_rem_^a^	*M*_sat_^a^
	°C	nm	10^19^ cm^−3^	cm^2^/*(V· s*)	Oe		

GM1	100	120	0.8	110	250	1.4 μ_B_	1.8 μ_B_
GM2	100	120	9.3	110	260	2.1 μ_B_	3.0 μ_B_
GM3	200	130	23	80	260	1.7 μ_B_	3.0 μ_B_
GM4	200	135	14	71	115	2.1 μ_B_	3.2 μ_B_
GM5	300	120	4.8	74	265	0.6 μ_B_	2.5 μ_B_

^a^Magnetic parameters were obtained for magnetic field oriented parallel to the sample plane; the magnetization values were calculated per manganese atom.

**Figure 1:**
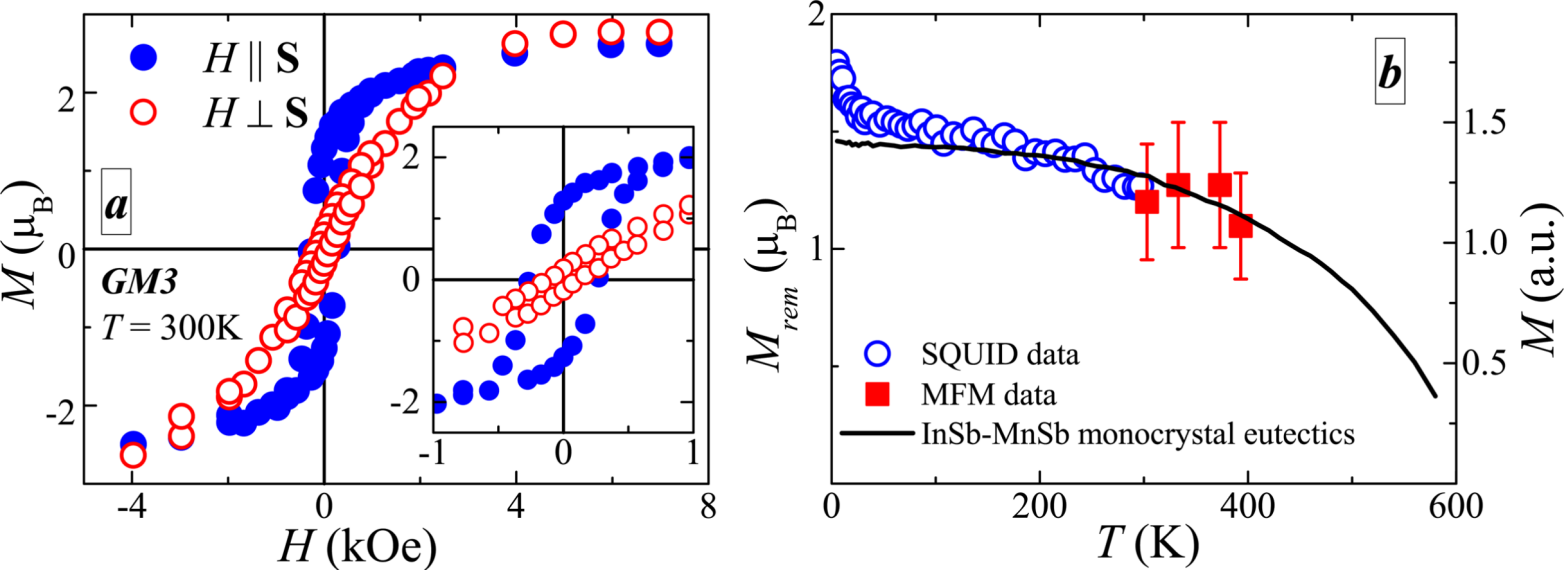
(a) Magnetization as a function of the magnetic field for sample GM3 at *T* = 300 K. Measurements were performed with the magnetic field oriented parallel to the sample plane (solid symbols) and perpendicularly to it (open symbols). The inset shows the hysteresis loop at low fields. (b) Temperature dependence of the remanent magnetization. Open circles are SQUID data for sample GM3, red squares are MFM data for the same sample and the black curve shows the temperature dependence of the saturation magnetization of the InSb–MnSb eutectic [17].

Figure 1a shows typical curves of the magnetization as function of the magnetic field for the sample GM3 (see Table 1) for magnetic field orientations parallel to the sample plane and perpendicularly to it. The presence of a well-pronounced hysteresis suggests that ferromagnetic ordering in these materials appears at *T >* 300 K. This is more evident from the temperature dependence of the remanent magnetization *M*_rem_ presented in Figure 1b. In the same figure we also provide the MFM data and temperature dependence of the remanent magnetization for InSb–MnSb eutectics. Nonzero *M*_rem_ values persist up to 400 K and the temperature dependence is close to that of the InSb–MnSb eutectic composition, which corresponds to the *T*_c_ value of MnSb of ca. 600 K [17]. Thus, presented data clearly proves the presence of high-temperature ferromagnetism in the samples under study.

As mentioned above, the studied system differs from traditional DMS materials by substantially higher carrier mobilities and *T*_c_ values, while the nature of the high-temperature ferromagnetism is not completely clear. Previous studies [9] suggested that ferromagnetism in (GaSb)_1−_*_x_*(MnSb)*_x_* is related to the interaction of charge carriers with MnSb nanoinclusions with *T*_c_ = 600 K [17–18]. This interaction is affected by the appearance of Schottky barriers at the MnSb/GaSb boundaries. Basically, these type of barriers appear on the semiconductor/metal interfaces providing a tunneling charge transfer across the boundary, if the barrier is high enough. In the present case, Schottky barriers may appear only if inclusions are sufficiently large to establish a second electronic phase (metal-type) despite the impact of the surrounding GaSb matrix. The parameters of these barriers (e.g., the width) substantially depend on the charge distribution, i.e., they depend on the carrier concentration.

It is worth mentioning that in real samples not all Mn atoms are strictly positioned in MnSb inclusions, some of the Mn atoms can be distributed within the semiconductor matrix. This results in a two-phase magnetic subsystem in which lower *T*_c_ values correspond to isolated Mn atoms within the matrix [19]. Also, as it is shown in Table 1, the *M*_sat_ values reach 3.2 μ_B_ per Mn atom, which is close to 3.6 μ_B_ obtained from the experimental data for MnSb samples analyzed earlier [18,20], but is lower than the value expected for Mn^2+^. This difference can be related to the presence of Mn^3+^ ions and antiferromagnetic interactions between carriers and Mn atoms.

**Figure 2:**
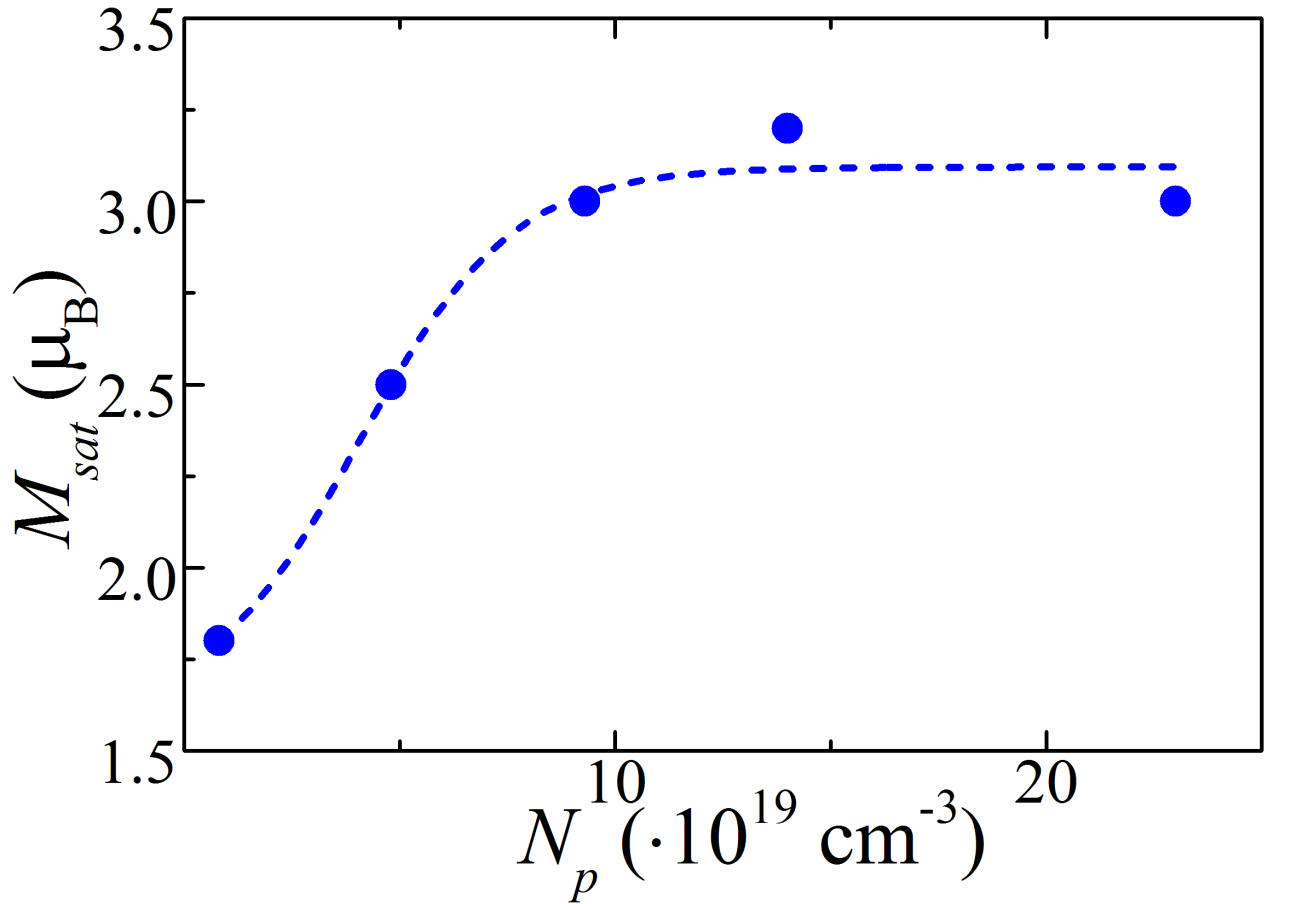
Room-temperature saturation magnetization (*M*_sat_) as a function of the carrier concentration (*N*_p_) (the dashed line is a guide to the eye).

One major parameter of the interaction of holes with MnSb inclusions is the width of the Schottky barriers *d*_barrier_, which decreases with increasing carrier concentration [9]. This picture correlates with the concentration dependence of the saturation magnetization *M*_sat_ presented in Figure 2. This also agrees well with the data obtained for previously studied (GaSb)_1−_*_x_*(MnSb)*_x_* films (see Figure 5 in [12]). As it can be seen from these figures, *M*_sat_ increases with carrier concentration and saturates above *N*_p_ ≈ 10^20^ cm^−3^ at which *d*_barrier_ becomes comparable with the effective penetration depth of the carrier (hole) wave function, *l*_p_, under triangular barrier. Taking into account that the energy gap in GaSb is *E*_g_ = 0.7 eV and the Schottky barrier height is about (1/3)*E*_g_[21], the value of *d*_barrier_ can be estimated as 2 nm at *N*_p_ = 10^20^ cm^−3^ while *l*_p_ is of the same value [9].

As shown in Figure 2, the values of *N*_p_ for the studied samples are sufficiently high to provide an effective interaction of holes with the MnSb inclusions and cause the saturation of magnetization. This, along with high *T*_c_ values, suggests that this interaction could be the main source of the high-temperature ferromagnetism in the studied systems. However, to justify the applicability of a model of interaction between carriers and inclusions, one needs to verify specific properties of spin-polarized system, for instance through transport measurements.

**Figure 3:**
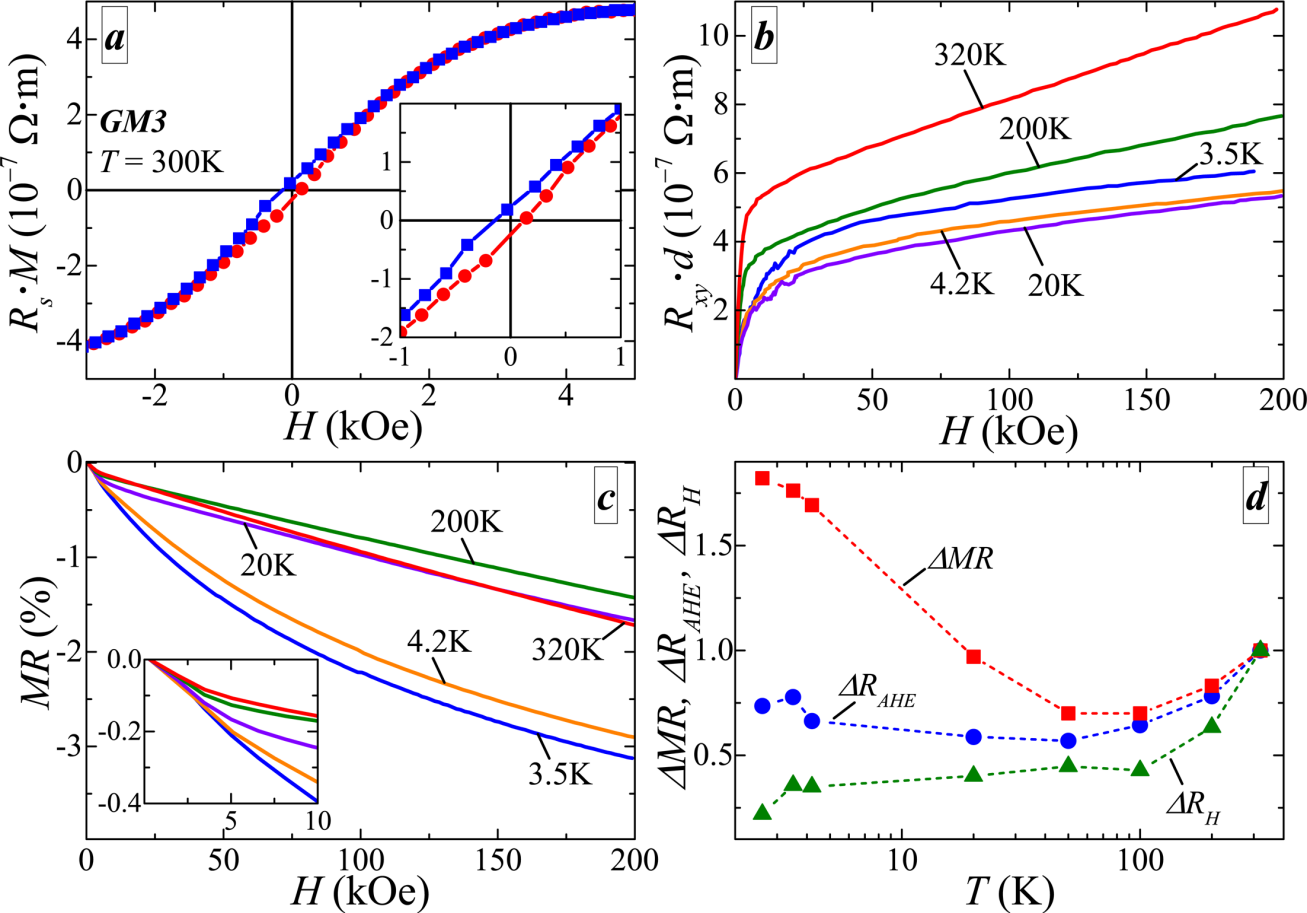
Magnetotransport properties of sample GM3: (a) Field dependence of the anomalous Hall component at 300 K (the linear background is subtracted). The slope of the curve correlates with the sign of the charge carriers (holes), i.e., the observed AHE is positive. The inset demonstrates hysteresis behaviour at low fields. (b) Field dependence of the Hall resistance up to *H* = 200 kOe at various temperatures. (c) Magnetoresistance at various temperatures. The inset shows the low-field part of the presented curves. (d) Temperature dependence of the high-field magnetoresistance ΔMR (at *H* = 200 kOe), saturated AHE amplitude Δ*R*_AHE_ and Hall slope Δ*R*_H_ normalized by the corresponding values at *T* = 320 K.

It should be noted that the mobility values have no pronounced dependence on the carrier concentration (see Table 1), i.e., the conductivity of the studied films is affected by various factors. Thus, magnetotransport phenomena can yield additional information about the system. The results of Hall resistivity measurements for the GM3 sample are shown in Figure 3a and Figure 3b. The Hall resistivity in magnetic systems can be divided into two parts:

[1]
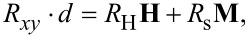


where *R*_H_ is the Hall constant, used for the determination of *N*_p_ and μ (see Table 1), and *R*_s_ is the anomalous Hall constant, which is defined by several parameters of the system [22]. Thus, the *R_xy_*(*H*) should reproduce the field dependence of the magnetization. As shown in Figure 3a, *R*_s_·*M*(*H*) (the Hall resistivity after subtraction of the linear contribution) demonstrates hysteresis behavior (see inset) with *H*_c_ ≈ 140 Oe and a saturation field value of *H*_sat_ ≈ 3.8 kOe. This is in a good agreement with values of *H*_c_ ≈ 130 Oe and *H*_sat_ ≈ 4 kOe obtained from SQUID data with the magnetic field oriented perpendicularly to the sample plane. Note, that the values of *H*_c_ and *H*_sat_ in Table 1 were obtained with the magnetic field oriented parallel to the sample plane. The difference between the values in Table 1 and the parameters obtained from the *R_xy_*(*H*) curves is related to a substantial magnetic anisotropy of the samples under study (see Figure 1). The observation of the anomalous Hall effect (AHE) clearly suggests that delocalized holes interact with the magnetic subsystem, i.e., that there are spin-polarized carriers even at room temperature. Thus, the exchange interaction in the studied system can be mediated by these spin-polarized holes as it was suggested earlier.

The combination of the experimentally observed high-temperature ferromagnetism and the spin-polarization of conducting carriers makes the studied films very promising for various applications. In order to obtain a deeper insight into the properties of these films, we have made additional magnetotransport measurements in high magnetic fields. Hall resistivity measurements in magnetic fields up to *H* = 200 kOe at various temperatures are presented in Figure 3b. It is clearly seen that at room temperature the anomalous Hall effect contribution saturates below *H* = 10 kOe and *R_xy_*(*H*) becomes linear. The linear slope and the saturation field are different for different temperatures. To visualise the temperature evolution of the presented curves we used a simple linear fit of the high field region. In this case the fitting function has two variables, the slope (*R*_H_) and an offset, which corresponds to the saturated AHE amplitude *R*_AHE_. A second sign for the interaction between magnetic and conducting subsystems is the appearance of a negative magnetoresistance (nMR), which is usually ascribed to spin-dependent scattering. As it is shown in Figure 3c, the studied samples exhibit nMR that does not saturate up to 200 kOe in the studied temperature range. It should be noted, that at low fields the nMR has a similar form at all temperatures (see inset in Figure 3c), while at higher fields the shape of MR curves changes. In particular, above 20 K the nMR is linear above 20 kOe, while below 4.2 K the shape of the nMR is close to sublinear or logarithmic, which is more common for a spin-dependent scattering contribution [23–25].

Based on the assumption of a two-phase magnetic subsystem (MnSb inclusions and isolated Mn atoms) we can qualitatively describe the temperature evolution of the magnetotransport parameters presented in Figure 3d. The character of the dependencies of Δ*R*_AHE_ and ΔMR is rather similar. Hence, they should have the same nature. At 50 K we observe a local minimum in both curves. The increase above 50 K can be related to the presence of Schottky barriers. If both AHE and nMR correspond to the interaction with MnSb inclusions, then their amplitudes are defined by the tunneling intensity, which increases with temperature (due to the triangular shape of the barrier). Below 50 K another contribution becomes significant, the interaction with isolated Mn moments. From our data we cannot define the corresponding *T*_c_ value accurately because this interaction can be significant even above the ordering temperature [24–25], while the total magnetic moment of isolated Mn atoms can be substantially smaller than that of MnSb inclusions. The temperature dependence of Δ*R*_H_ is more complicated. But in the present case of a large AHE contribution, MR and AHE itself can strongly affect *R*_H_ through the relations of conductivity and resistivity tensors [26]. It should be noted that nMR at high temperatures can be also due to tensor relations, although the mentioned difference of the nMR functional form at various temperatures suggests that, at least at low temperatures, the nMR should be related to spin-dependent scattering. Also there are several other phenomena that can be relevant. Thus, to elucidate their contributions, a more detailed study with quantitative simulations is needed. However, this it is out of the scope of the present paper.

To establish high-temperature ferromagnetism of MnSb inclusions via spin-polarized carriers their interaction have to be sufficiently strong. This implies that has to be a large amount of such inclusions with distances between them shorter than the carrier spin-relaxation length. However, the comparatively high carrier mobilities in the studied samples still leave some doubt on the mentioned idea, because a great number of MnSb inclusions (as well as a high concentration of isolated Mn atoms) should induce intense scattering of carriers, i.e., low mobilities. The latter, according to estimates, should not exceed 10 cm^2^/(V·s), as it is in GaMnAs [27] and in the previously studied GaMnSb compound with the highest *T*_c_ [9]. This contradicts the suggestion that MnSb nanoinclusions are distributed over the whole volume of the film. Thus, a detailed knowledge of samples structure is required to resolve this problem properly. To get this information we performed additional measurements using S/TEM, AFM and MFM methods.

**Figure 4:**
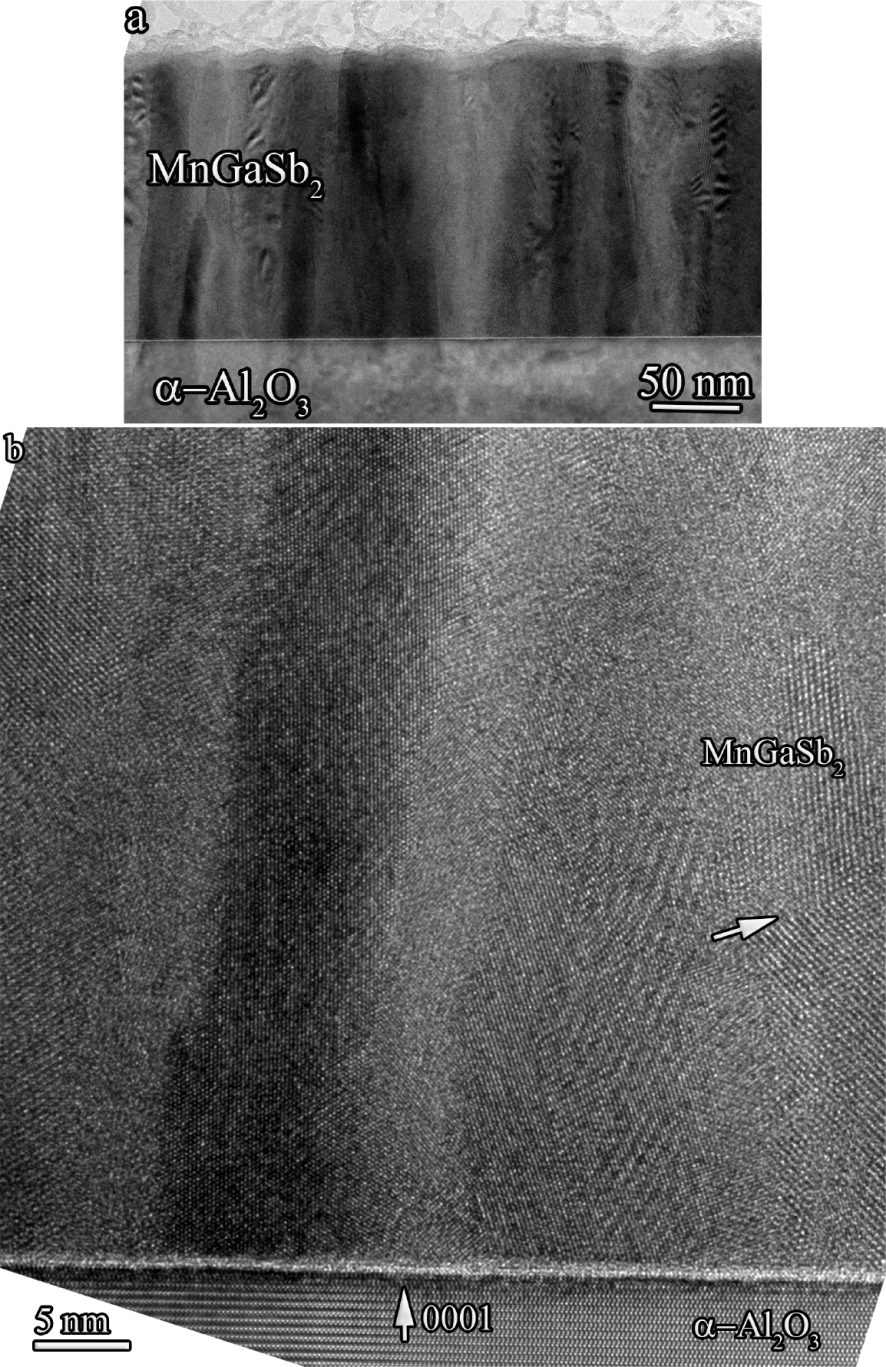
TEM images of the film cross section after annealing (sample GM3): (a) bright-field image, (b) HRTEM image.

In the present TEM/EDX microanalysis study of the GaSb–MnSb/α-Al_2_O_3_ system the actual investigation was performed on a cross-sectional piece of sample GM3 with lateral sizes of about 1 μm. The results are presented in Figure 4a and Figure 4b. The film thickness is about 150 nm and the surface roughness does not exceed 6 nm. This is in a good agreement with the data presented in Table 1, which were estimated from the duration of the deposition process. The image analysis confirms a homogeneous composition throughout the film thickness without any substantial contrast variations. Contrast changes in the lateral direction are due to diffraction contrast arising from the columnar film microstructure, which was distinctly observed in bright-field TEM (Figure 4a) and in high-resolution bright-field TEM (HRTEM) images (Figure 4b), and even affects the HAADF TEM image (not presented here).

**Figure 5:**
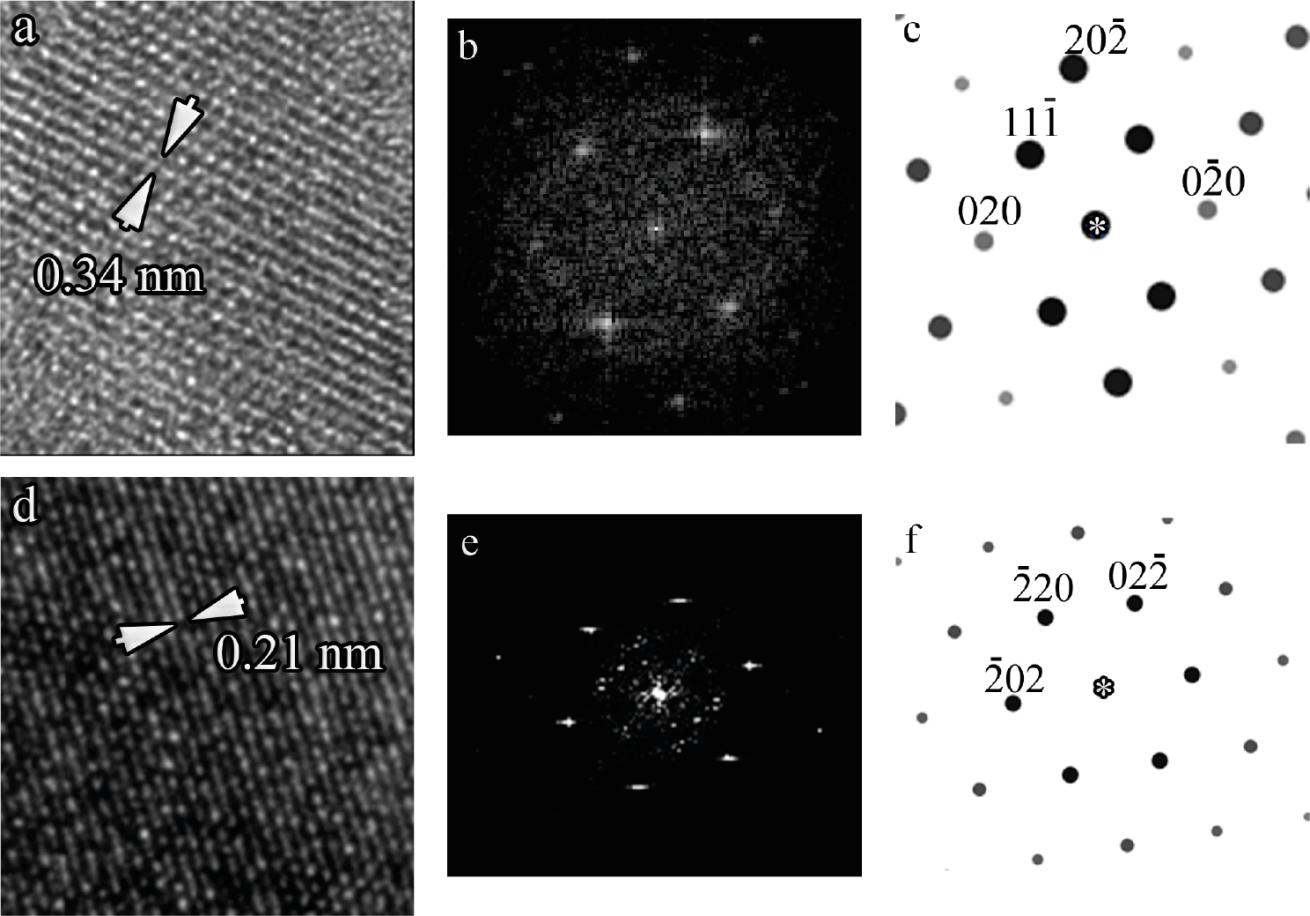
(a,d) HRTEM images of sample areas. (b,e) Corresponding two-dimensional Fourier spectra. (c,f) Calculated electronograms of the cubic MnGaSb_2_ compound in [101] (c) and [111] (f) projections.

Energy-dispersive X-ray microanalysis (EDX) of the film composition near the interface edge and at a distance from it yielded the ratio Mn/Ga/Sb = 30:30:40 with 2% accuracy. A HRTEM image of studied film is presented in Figure 4b. Fast Fourier-transform (FFT) analysis of the high-resolution image areas in two directions (Figure 5b and Figure 5e) and direct analysis of the crystal cell image (Figure 5c and Figure 5f) were used to analyze the crystalline structure of the sample. As an example, the results for two of ten studied image areas are presented in Figure 5.

The analysis of all studied areas unambiguously indicates that the structure of the film is cubic and corresponds to the GaSb crystal structure (space group 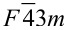). Moreover, the images clearly show twins and stacking faults, which are typcal for cubic GaSb crystals. At the same time, the electron diffraction data obtained for pristine films correspond to diffraction patterns for the hexagonal compound with the space group *P*6_3_/*mmc*. Also, the image analysis showed that the morphology of the film and the lateral dimensions of the film columns remained the same after annealing. It should be noted, that the energy-dispersive X-ray microanalysis showed a slight (2–3%) decrease of Mn content in the film volume after annealing. This can be related to the details of sample preparation (e.g., the presence of a thicker damaged layer on the surface) or to the diffusion of Mn atoms. Thus, we can conclude that film annealing causes a phase transition of the hexagonal GaSb matrix to a cubic matrix.

The electron microscopy data unambiguously shows that the columnar microstructure of the film persists up to almost the surface layers. It should be noted, that no signs of second-phase precipitates in the film volume was observed. In particular, using a GaSb–MnSb eutectic composition as a target for sputtering allowed us to avoid the formation of MnGa inclusions, which were observed earlier in samples obtained by laser deposition from Mn and GaSb targets, without taking the stoichiometry into account [28].

**Figure 6:**
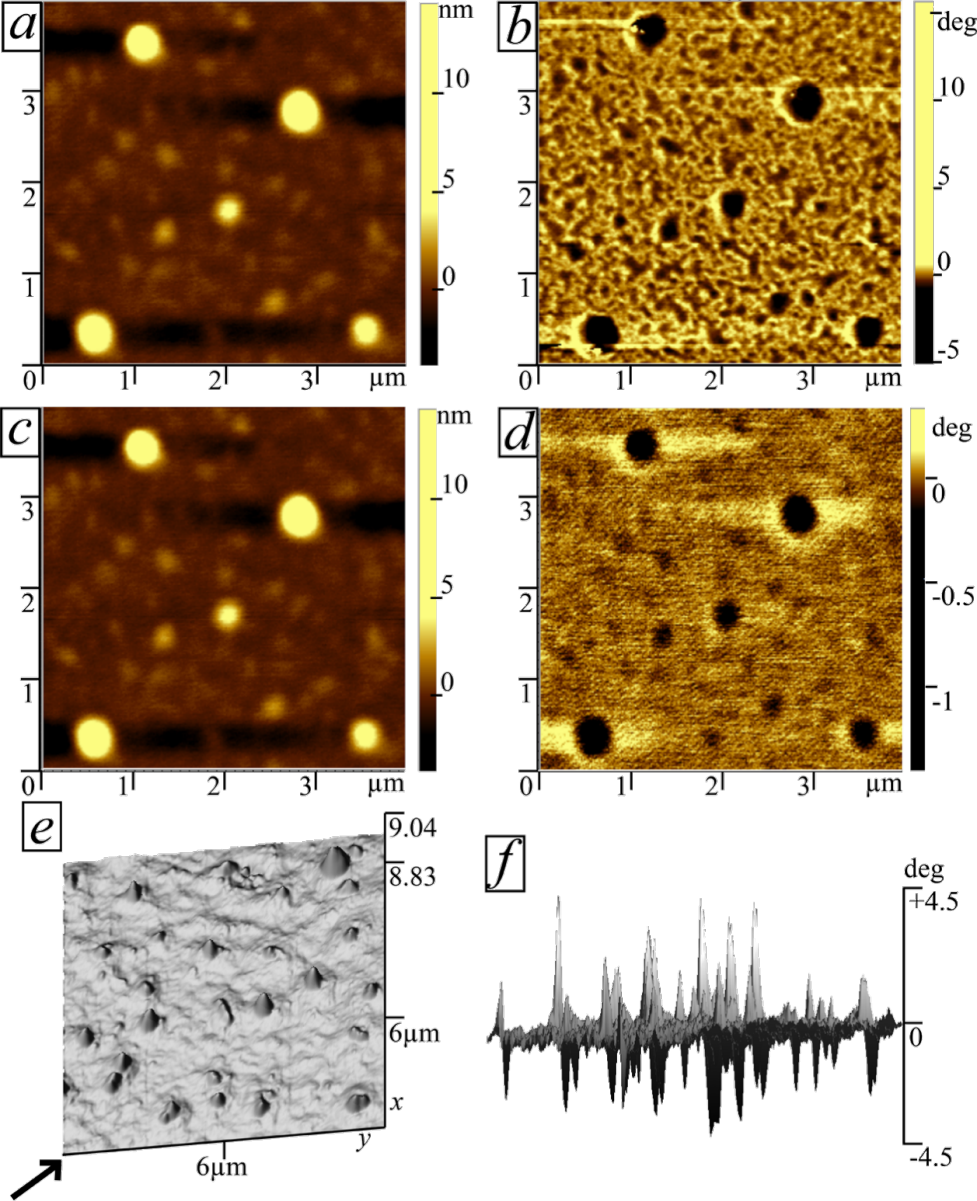
AFM (a,c) and MFM (b,d) images of the same surface at 303 K (a,b) and 413 K (c,d). AFM (e) and MFM (f) three-dimensional image of the surface containing ferromagnetic inclusions. The arrow in (e) corresponds to the direction for which MFM image (f) was made.

However, due to unevenness of the film and due to oxidation, the top layer of the film could not be studied carefully and we cannot determine the exact morphology and composition of the surface. Hence, AFM and MFM measurements were performed. AFM and MFM images of one sample are presented in Figure 6. The increase of temperature from 303 to 413 K does not change surface topology containing regions of larger height (Figure 6a and Figure 6c), while the phase contrast in the MFM images (Figure 6b and Figure 6d), which characterizes magnetization of the film, decreases at higher temperatures. Nevertheless, the MFM images reveal the presence of a considerable phase contrast even at 413 K, which means that the sample exhibits ferromagnetism with *T*_c_ much higher than room temperature. As it was mentioned before, high *T*_c_ values suggest that observed surface imperfections are MnSb inclusions. A comparison of the AFM and MFM data shows that magnetic moments are distributed mostly within areas of larger height (MnSb inclusions) detected by AFM. Magnetic inclusions were detected over the whole film surface. A three-dimensional AFM image of another surface region at 303 K is shown in Figure 6e. The corresponding MFM image (Figure 6f) demonstrates the presence of sub-micrometer ferromagnetic inclusions with aligned magnetic poles. Combining these results with SEM and TEM data suggests that MnSb inclusions are ferromagnetic and located mostly close to the film surface, rather than being evenly distributed within the sample volume. This assumption also provides a good explanation for the high mobility values of conducting holes, since magnetic inclusions localized on the surface have a substantially weaker effect on scattering in studied films.

## Conclusion

We have studied structural, magnetic and magnetotransport properties of annealed (GaSb)_1−_*_x_*(MnSb)*_x_* films with *x* = 0.41 and thickness *d* = 120–135 nm. Electron microscopy data suggests that studied films have single-phase columnar structure in the volume. It was found that annealing process induces a phase transition of hexagonal GaSb matrix into a cubic matrix. AFM and MFM studies revealed the presence of ferromagnetic MnSb inclusions near the surface of the films. This perfectly explains the high mobility values of charge carriers in these systems. Experimental observation of these inclusions is crucial for the explanation of high-temperature ferromagnetism, since the magnetization hysteresis at room temperature perfectly fits the idea of a dominant contribution of MnSb inclusions with *T*_c_ *>* 400 K interacting with holes. This is confirmed by the appearance of a substantial anomalous Hall effect even at room temperature that signifies the presence of spin-polarized charge carriers. The interaction of charge carriers with inclusions is greatly affected by the Schottky barriers at GaSb/MnSb boundaries. Due to high carrier concentrations in the studied films, the transparency of these barriers is rather high, which explains the saturation of magnetization at *N*_p_ = 10^20^ cm^−3^.

## Acknowledgements

This work was partially supported by Russian Foundation for Basic Research (grants #17-02-00262 and #16-03-00150) and by Ministry of Education and Science of Russian Federation (grant #16.2814.2017/PCh).
